# Intestinal stem cells in intestinal homeostasis and colorectal tumorigenesis

**DOI:** 10.1093/lifemedi/lnae042

**Published:** 2024-12-25

**Authors:** Gaoli Shi, Yang Li, Haihong Shen, Qiankun He, Pingping Zhu

**Affiliations:** School of Life Sciences, Zhengzhou University, Zhengzhou 450001, China; School of Life Sciences, Zhengzhou University, Zhengzhou 450001, China; School of Life Sciences, Gwangju Institute of Science and Technology, Gwangju 61005, Republic of Korea; School of Life Sciences, Gwangju Institute of Science and Technology, Gwangju 61005, Republic of Korea; School of Life Sciences, Zhengzhou University, Zhengzhou 450001, China; School of Life Sciences, Zhengzhou University, Zhengzhou 450001, China

**Keywords:** intestinal stem cells, cancer stem cells, self-renewal, differentiation, niche

## Abstract

Colorectal cancer (CRC), one of the most common tumors in the world, is generally proposed to be generated from intestinal stem cells (ISCs). Leucine-rich repeat-containing G protein-coupled receptor 5 (Lgr5)-positive ISCs are located at the bottom of the crypt and harbor self-renewal and differentiation capacities, serving as the resource of all intestinal epithelial cells and CRC cells as well. Here we review recent progress in ISCs both in non-tumoral and tumoral contexts. We summarize the molecular mechanisms of ISC self-renewal, differentiation, and plasticity for intestinal homeostasis and regeneration. We also discuss the function of ISCs in colorectal tumorigenesis as cancer stem cells and summarize fate dynamic, competition, niche regulation, and remote environmental regulation of ISCs for CRC initiation and propagation.

As the major organ for nutrient absorption and a key barrier against the external stimulus, the intestine contains epithelium and lamina propria. Intestinal epithelium contains two lineages: absorptive cells and secretory cells. Absorptive lineage cells, account for 80%–90% of all differentiated cells, contain enterocytes and microfold (M) cells, and enterocytes are mainly responsible for absorbing nutrients and water; whereas M cells recognize and transport antigens in the cavity to the underlying Peyer’s patches for immune response/tolerance. Secretory lineage cells include goblet cells that secrete mucus, Paneth cells that secrete antimicrobial peptides, Tuft cells that mediate chemical sensing, and enteroendocrine cells that produce hormones. Intestinal cells are in a complicated, dynamic, and harsh environment, suffering from physical peristalsis, variable pH, various digestive enzymes, and a variety of bacterial microorganisms. Intestinal epithelium is the fastest-renewing tissue in adult mammals, turning over every 3–5 days. Most mature intestinal epithelial cells only survive for a few days except for Paneth cells, which survive 1–2 months. This rapid turnover relies on the powerful self-renewal capacity of ISCs [1], and the active migratory forces of epithelial cells revealed recently [2].

## Intestinal stem cells

Identification of Intestinal stem cells (ISCs) requires marker verification and lineage tracing. In recent years, multiple markers of ISCs have been identified, including Lgr5 [3], Olfactomedin 4 (Olfm4) [4], Achaete scute-like 2 (Ascl2) [5], B-cell-specific Moloney murine leukemia virus integration site 1(Bmi1) [6], mouse telomerase reverse transcriptase (mTert) [7], Leucine-rich repeats and immunoglobulin-like domains 1 (Lrig1) [8] and so on. Among them, Lgr5, Olfm4, and Ascl2 are markers of crypt base columnar cells (CBCs) which are in a proliferative state at the bottom of the crypt, and Olfm4 serves as ISC marker for murine small intestine, human small intestine, and colon, but not colon of mice. Bmi1 and mTert are markers of “+4” cells which are in a quiescent state, and quiescent Lrig1^+^ stem cells are also at the bottom of the crypt. However, through lineage tracing assay of label-retaining cells, “+4” stem cells are recognized as descendants of Lgr5^+^ ISCs and are precursors of Paneth cells and enteroendocrine cells [9]. Taking advantage of R-spondin (RSPO) responsiveness and single-cell sequencing of intestinal crypts and VIPER algorithm analysis, the latest two studies reveal that true ISCs are located in the isthmus of the intestinal crypts, with high expression of Fibroblast growth factor binding protein 1 (*Fgfbp1*) and TNF receptor superfamily member 19 (*Tnfrsf19, Troy*), respectively, while Lgr5^+^ stem cells are progeny cells of these true ISCs [10, 11].

ISCs harbor many characteristics. In an excellent review, Snippert and colleagues divide these characteristics into phenotype, activity, potential, and functionality. ISC phenotype refers to cell morphology and markers. ISC activity encompasses pluripotency and self-renewal ability, often assessed by lineage tracing. ISC potential refers to all cells with stemness in special scenarios such as damage and regeneration. In contrast to activity and potential, which describe the intrinsic characteristics of ISCs, ISC functionality refers to the characteristics of all stem cell populations within the crypt. While the contribution of a single ISC to the functional compartment cannot be determined, ISCs closer to the bottom of the crypt are more likely to produce lineages [12]. In this review, we focus on describing the self-renewal, differentiation, and plasticity of ISCs, which are also some classic characteristics of these cells.

### ISC self-renewal

Like many other stem cells, ISCs have the ability to self-renew and differentiate. The fate of ISCs is mainly determined by a variety of signals, including RSPO/Lgr, WNT/β-catenin, Notch, epidermal growth factor (EGF), bone morphogenetic protein (BMP), Hippo/Yap, and so on. The balance between these activating signals, such as RSPO/Lgr and WNT/β-catenin, and inhibitory signals, such as BMP, synthetically determines the fate of ISCs [13]. Most of these signals are generated by epithelial, mesenchymal, and immune cells in the microenvironment, which determine the microenvironmental zone of ISCs by providing various and redundant niche factors. ISCs compete for microenvironmental space and drift neutrally, and cells pushed out of the stem cell zone differentiate immediately [14, 15].

**Figure 1.**
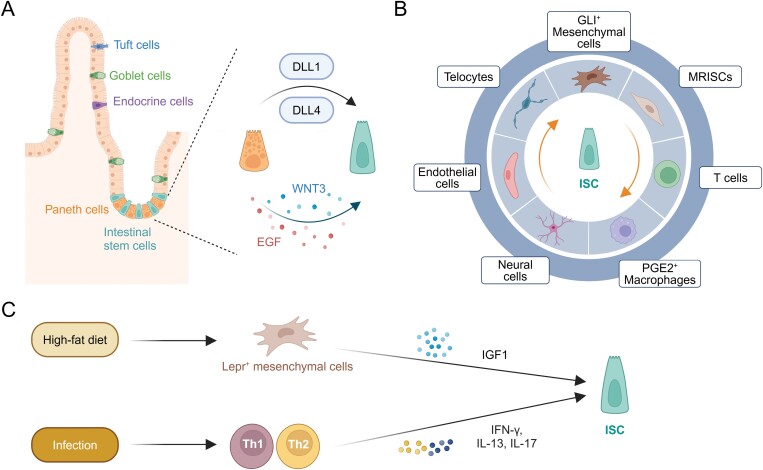
**ISCs and their niche cells.**The self-renewal of ISCs is finely regulated by various niche cells, including epithelial cells (A) and non-epithelial cells (B), and ISC niche is dynamic (C). The figures were created with BioRender.com.

Epithelial Paneth cells are the most classic type of ISC niche cells. As the direct neighbor cells of ISCs, Paneth cells contain a developed endoplasmic reticulum and Golgi system to secrete WNT3 and EGF to maintain ISC self-renewal; meanwhile, Notch ligands Delta-like 1 (DLL1) and DLL4 highly expressed on the membrane of Paneth cells activate the Notch signaling in ISCs (Fig. 1A) [16]. However, the effect of Paneth cells elimination on ISC maintenance is controversial: classically, it was found that the reduction of Paneth cells, through Growth factor independent 1 transcriptional repressor (*Gfi1*) knockout, CR2-tox176, or SRY-Box transcription factor 9 (*Sox9*) knockout, triggered a reduction in Lgr5^+^ ISCs, and the remaining Paneth cells and Lgr5^+^ ISCs had a coincident distribution, conferring Paneth cells as the major niche cells of Lgr5^+^ ISCs *in vivo* [16]. However, extensive subsequent studies dispute that loss of Paneth cells does not cause ISC reduction in the *Atoh1* knockout mouse or pLysDTR mouse model, suggesting a functional redundancy of Paneth cells for ISCs maintenance [17, 18]. When Paneth cells are depleted, other cells expressing Notch ligand DLL1/DLL4, such as tuft cells and endocrine cells, replenish the loss of Paneth cells and promote the function of Lgr5^+^ ISCs [18]. In fact, the non-essential of Paneth cells may be hinted at in the spatiotemporal profiles between Paneth cells and ISCs: (1) During intestinal development, Lgr5^+^ ISCs appear earlier than Paneth cells, indicating they emerge via a Paneth cell-independent manner; (2) Unlike the small intestine, there are no Paneth cells in the colon. ISCs in the colon are distributed separately from REG4^+^ secretory cells, which are located deep in the crypt and highly express DLL1/DLL4 to activate the Notch signaling pathway of ISCs. However, REG4^+^ cells do not express Wnt, indicating that other cells provide Wnt for ISC self-renewal [19].

In addition to Paneth cells, ISCs are surrounded by stromal cells, immune cells, and neurons (Fig. 1B), accumulating studies reveal that these microenvironmental cells promote the maintenance and self-renewal of ISCs by producing WNT, RSPO, and BMP inhibitor Noggin. Among them, Forkhead Box L1 (FOXL1)-positive telocytes are in closely contact with intestinal epithelium and secrete stem factors such as Wnt2b and Rspo3 to promote the self-renewal of ISCs. Porcupine (*Porcn*) knockout in telocytes impairs WNT/β-catenin activation in ISCs, reducing ISC numbers and destructing intestinal structure [20]. In the large intestine lacking Paneth cells, GLI1^+^ mesenchymal cells are key niche cells for Lgr5^+^ ISCs. Deletion of Wntless (*Wls*), a mediator of Wnt ligand secretion, in GLI1^+^ cells leads to a reduction of ISC number and a disordered structure in colon tissue, with no effect in the small intestine. In the small intestine, Lgr5^+^ ISCs reduction and structural disorder are only observed upon *Wls* knockout in GLI1^+^ and Villin^+^ cells simultaneously. During intestinal regeneration after dextran sodium sulfate (DSS)-induced damage, GLI1^+^ mesenchymal cells are enriched around the colon crypts to meet the requirement for ISCs [21]. Bing Su and colleagues identify MAP3K2-regulated intestinal stromal cells (MRISCs) as ISC niche cells, which are located at the base of colon crypts and are a novel subset of CD90^med^GP38^+^ stromal cells. MRISCs are the main source of RSPO1 during DSS treatment and play a key role in maintaining the number and function of colon ISCs during DSS damage [22]. ISCs are rapidly proliferative and highly express MHC and TAP proteins, and thus are vulnerable to T cells, which may be the reason why the intestine is prone to graft-versus-host reaction (GVHR) [23]. Tissue-resident immune cells also serve as niche cells for ISCs. For example, prostaglandin E2 (PGE2)-positive macrophages maintain the self-renewal of ISCs by secreting PGE2 [24]. Recently, it has been discovered that neurons [25, 26] and lymphatic endothelial cells [27–30] are also important cell components in the ISC niche.

Interestingly, the ISC niche is dynamic along with the changes in external environment, such as dietary environment and infection. Leptin receptor (Lepr)-positive mesenchymal cells exist in the ISC niche and respond to dietary status dynamically, with an increased cell number in a high-fat diet and a decreased number in fasting. Lepr^+^ mesenchymal cells promote the rapid self-renewal of ISCs by secreting insulin-like growth factor 1 (IGF1) (Fig. 1C) [31]. This elegant work linked high-fat diet and ISCs through a kind of mesenchymal cells in the ISC niche, revealing an additional layer to direct crosstalk between high-fat diet and ISCs [32]. During intestinal infection, MHC^+^ ISCs show robust MHC expression and emerge as non-classical antigen-presenting cells. Meanwhile, activated Th1, Th2, and Th17 cells promote the differentiation into the secretory lineage of ISCs at the expense of self-renewal, by IFN-γ, IL-13, and IL-17. On the contrary, Treg cells, generally enriched in the late stage of infection, promote the self-renewal of ISCs through IL-10, to supplement ISCs which are reduced in early infection (Fig. 1C) [33].

### ISC differentiation

As mentioned above, intestinal epithelium contains secretory lineage and absorptive lineage. The cell numbers and proportion of these two lineages are strictly controlled, which is mainly determined by the directed differentiation of ISCs. Signaling pathways such as WNT, Notch, and EGF play a key role in ISC differentiation [34]. Notably, these signals can be manipulated in organoids to enrich specific cell types, further validating the key role of these signals in the directed differentiation of ISCs [35]. Among them, Hairy and enhancer of split 1 gene (HES1)/ATOH1 (also known as MATH1)-mediated DLL1/Notch lateral inhibition plays a core role in the directed differentiation of ISCs, controlling the differentiation either into absorptive progenitor cells or secretory progenitor cells.

**Figure 2.**
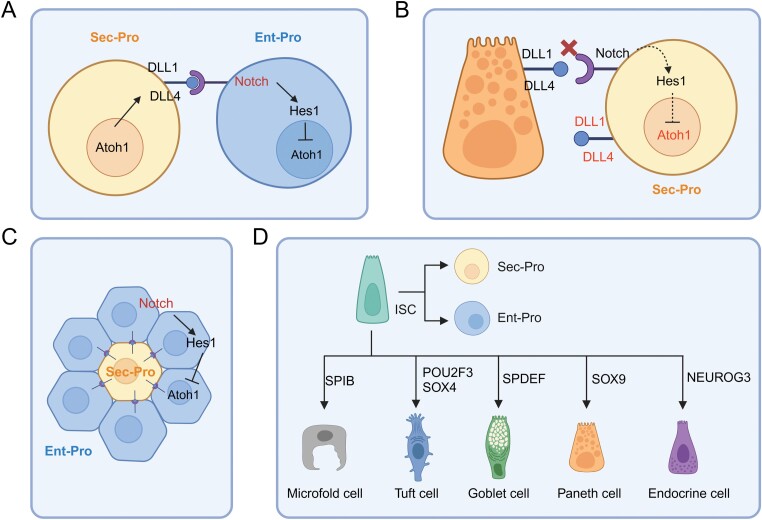
**Molecular mechanism of ISC differentiation.**Lateral inhibition (A–C) determines absorptive/secretory lineages and transcription factors determine terminal differentiation (D). The figures were created with BioRender.com. ‘Sec-Pro’ refers to secretory progenitor cells and ‘Ent-Pro’ refers to absorptive enterocyte progenitor cells.

The core mechanisms of DLL1/Notch lateral inhibition include: ATOH1 is a master transcription factor of secretory progenitor cells and induces DLL1/DLL4 expression, which activates the Notch pathway in neighbor cells directly contacted to ATOH1^+^ cells, and in turn these neighbor cells, exhibiting HES1 activation and ATOH1 inhibition, are fate-determined into absorptive progenitor cells (Fig. 2A) [36]. As shown in Fig. 2B, when daughter cells leave the ISC zone, due to the loss of DLL1/DLL4 support from Paneth cells, the Notch–HES1 signaling is blocked, ATOH1 is activated, and DLL1/DLL4 is expressed, finally, these cells are fated into the secretory lineage. With DLL1/DLL4 expression, secretory progenitor cells support the activation of Notch signaling in 6–8 directly-contacted cells, inhibiting ATOH1 expression via HES1, so that these neighbor cells differentiate into absorptive lineage cells (Fig. 2C). Only if the surrounding 6–8 cells are all absorptive lineage, a progenitor cell will differentiate into secretory lineage as lose of DLL1/DLL4 supporting. This process, termed lateral inhibition, ensures a fixed ratio of secretory lineage and absorptive lineage in intestinal epithelium, and also serves as a molecular basis of the fact that intestinal absorptive cells are much more than secretory cells. Lateral inhibition has been validated by multiple assays, including various NOTCH-related inhibitors and knockout mice. For example, *Hes1* knockout induces all epithelial cells to be secretory cells [37], while *Atoh1* knockout leads to the loss of secretory cells thoroughly [38].

Besides lateral inhibition of Notch signaling to determine absorptive/secretory lineage, ISC differentiation is also precisely regulated at multiple other levels, especially transcription factors for terminal differentiation into specific cells, for example, Spi-B transcription factor (SPIB) for the differentiation into M cells, POU class 2 homeobox 3 (POU2F3)/SRY-Box transcription factor 4 (SOX4) for tuft cells, SAM pointed domain containing ETS transcription factor (SPDEF) for goblet cells, SOX9 for Paneth cells, and neurogenin 3 (NEUROG3) for enteroendocrine cells (Fig. 2D). For more details, the readers can refer to a relevant excellent review [13].

### ISC plasticity

**Figure 3.**
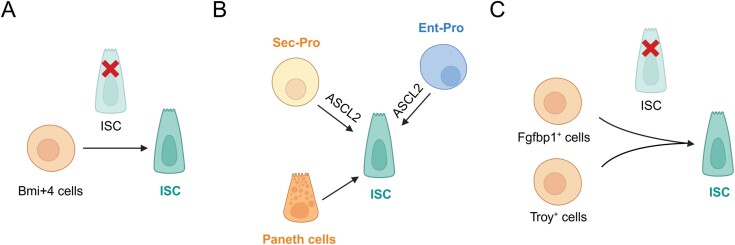
**The plasticity of ISCs.**Upon ISC depletion, new ISCs are dynamically generated from “+4” reserve stem cells (A), precursor cell dedifferentiation (B), or multipotent ISCs repopulation (C). The figures were created with BioRender.com.

Lgr5^+^ ISCs play a key role in intestinal homeostasis. However, a pioneering study revealed that treatment of *Lgr5*^DTR-EGFP^ mice with diphtheria toxin for 10 days completely destroyed Lgr5^+^ ISCs without influence on intestinal structure. Further analysis demonstrates that Bmi^+^ “+4” stem cells are able to replenish the lost Lgr5^+^ stem cells and serve as reserve stem cells [39]. This work raised the first model to explain intestinal maintenance after the elimination of Lgr5^+^ ISCs: reserve stem cells (Fig. 3A). Subsequently, a dedifferentiation model from progenitor or terminally differentiated cells into Lgr5^+^ ISCs has been discovered, including absorptive progenitor cells [40], secretory progenitor cells [41] and Paneth cells [42]. The vast extent of dedifferentiation is critical for maintaining intestinal homeostasis upon elimination of Lgr5^+^ ISCs. Tamoxifen treatment of *Lgr5*^GFP-CreERT2^;*Rosa26*^lsl-tdTomato^ mouse models followed by radiation damage reveals that almost all ISCs eliminated by radiation damage are replenished by dedifferentiation of absorptive and secretory progenitor cells, while reserve stem cells play little roles. These recent ISC progenies are still expressing ISC marker ASCL2, which drives the dedifferentiation into Lgr5^+^ ISCs through ASCL2–IL11Ra1 pathway (Fig. 3B) [43]. Recently, two studies proposed a third model: in the rapidly proliferating zone of the intestine or crypt isthmus, there is a group of true ISCs, which are characterized by high expression of Fgfbp1 or Troy and differentiate downward to produce Lgr5^+^ stem cells. Accordingly, it will not cause substantial damage when Lgr5^+^ stem cells are eliminated, while the elimination of Fgfbp1^+^ stem cells results in disordered intestine structure and impaired regeneration (Fig. 3C) [10, 11]. Although multiple compensatory mechanisms have been revealed after the elimination of Lgr5^+^ ISCs, some new findings rebut that Lgr5^+^ ISCs are indispensable in intestinal maintenance. For example, taking advantage of an updated *Lgr5*^2A-DTR^ mouse model, Nick Barker et al. demonstrate that Lgr5^+^ ISCs are of importance for the maintenance of intestinal epithelium *in vivo* and organoid propagation *in vitro*, pointing out two defects in the previous study [39]. (1) The expression levels of Lgr5/DTR in *Lgr5*^DTR-EGFP^ mice are much lower than that in *Lgr5*^2A-DTR^, leading to incomplete elimination of Lgr5^+^ ISCs upon DT treatment; (2) DT treatment should be repeated every day instead of once the second day, in which functional stem cells are not being completely blocked because of rapid plasticity and differentiation of ISCs [44]. Nevertheless, the well-accepted view is that Lgr5^+^ stem cells play an important but redundant role in intestinal homeostasis and regeneration. Extensive cell-fate remodeling occurs upon ISC deletion, including reactivation of reserve cells, and dedifferentiation of progenitor or mature cells, which further drives the regeneration process after intestinal damage.

During the injury and regeneration process, progenitor and mature cells not only dedifferentiate into ISCs, but also return to the fetal-like state, an interesting newly-discovered ISC state [45]. In early stage of intestinal development, ISCs are evenly distributed within the intestinal epithelium. During villus formation, morphological alternation induces an enrichment of Sonic hedgehog (SHH) signal at the villus tip, further establishing a “villus cluster” signaling center in the tip mesenchyme. The villus cluster BMP4 signal restricts ISCs to the bottom of the crypt through antagonism to WNT/β-catenin signaling [46]. The generation of villus curvature and villus clusters depends on the aggregation of platelet-derived growth factor receptor alpha (PDGFRA)-positive subepithelial stromal cells, the activation of matrix metallopeptidase 2 (MMP2) and its downstream extracellular matrix remodeling, while myosin heavy chain 9 (MYH9) and MYH10-regulated actomyosin activity is essential for PDGFRA^+^ stromal cells movement and villus generation [47]. By observing the proportion of Lgr5^+^ ISCs and their descendant cells at various developmental time points, Jordi Guiu and colleagues believe that Lgr5^+^ ISCs themselves are not sufficient for rapid growth during intestinal development. Taking advantage of CK19 (a widely expressed keratin)-inducible Cre and CK20 (a villus expressed keratin)-inducible Cre mice for lineage tracing, they demonstrate that both embryonic Lgr5^−^ and Lgr5^+^ cells generate adult Lgr5^+^ ISCs, and this phenomenon is related to the extensive epithelial remodeling during intestinal development, in which crypt fission and epithelial bending are basic for intestinal remodeling, allowing intestinal cells to reversibly switch between LGR5^−^ and LGR5^+^ states [48].

When the adult intestine is infected by *Heligmosomoides polygyrus*, the number of Lgr5^+^ ISCs decreases around granuloma which is formed by larvae-immune infiltration, while the number of spinocerebellar ataxia type 1 (Sca1)-positive fetal-like stem cells increases via an IFN-γ-dependent manner. *In vitro* organoid formation also exhibits a fetal-like phenotype, including large and smooth spheroids, and crypts lacking budding. In addition to parasitic infection, anti-TCR-activated T cell responses, radiation damage, and DSS damage also induce ISCs to return to a fetal-like state [49]. The study by Kim B. Jensen et al. demonstrates that the fetal-like state of colon ISCs induced by DSS damage is closely related to the remodeling of extracellular matrix, FAK/Src signaling, and activation of YAP/TAZ, which was verified through a collagen 3D organoid formation model [50]. Recently, single-cell sequencing of intestinal regeneration after radiation damage identified clusterin (Clu)-positive revival stem cells as damage-induced ISCs, which were rarely distributed in a steady state but expanded in a YAP1-dependent manner during the regeneration process [51].

Plasticity is essentially determined by the similarity of chromatin states between these cells, which have similar histone modifications and can effectively overcome chromatin barriers during remodeling. ISCs, absorptive progenitor cells, and secretory progenitor cells show extensive similarities at epigenetic levels, including DNA methylation, chromatin accessibility, and histone modifications. There are only 50 different DNA methylation regions between Lgr5^+^ ISCs and Lgr5^low^ progenitor cells, indicating that differentiation-related genes are already in a primed state in ISCs and progenitor cells are easily reprogrammed into ISCs [52]. Ramesh Shivdasani and colleagues collect secretory progenitor cells induced by dibenzazepine (DBZ) treatment or recombination signal binding protein for immunoglobulin kappa j region (*Rbpj*) knockout, as well as absorptive progenitor cells induced by *Atoh1* knockout, and reveal that absorptive and secretory progenitor cells harbor broadly similar chromatin accessibility, and the difference between these two lineages is mainly driven by transcription factors such as Atoh1. Meanwhile, these similarities also confer the lateral inhibition as a reversible process. Treatment with Notch inhibitors for more than 2 days induces ISCs into secretory lineage, and an inducible *Atoh1* knockout reverses these secretory lineage cells into absorptive cells. Thus, the broad similarity of chromatin accessibility provides a molecular basis for the dedifferentiation of mature cells into ISCs and the transdifferentiation between different lineages [53]. Taking advantage of RNA sequencing-based lineage trajectory analysis, they defined Bmi1^+^ and CD69^+^CD274^+^ cells as progenitor cells of endocrine cells and goblet cells and demonstrated that these progenitor cells dedifferentiated into Lgr5^+^ stem cells upon loss of ISCs. Moreover, Lgr5^+^ ISCs harbor similar histone modifications to Bmi1^+^ and CD69^+^CD274^+^ cells, indicating that the chromatin state plays a fundamental role in differentiation, dedifferentiation, and transdifferentiation [54].

### ISC and organoids

Organoids are *in vitro* 3D cultures that mimic the cell composition, structure, and function of an organ. In recent years, along with the intensive investigation of tissue stem cells such as ISCs, scientists have updated culture methods and obtained a deeper understanding of the molecular basis of organoid formation. Organoids provide important insights into the self-renewal, differentiation, regeneration, and molecular mechanism of ISCs [55]. Single Lgr5^+^ stem cells expand indefinitely in conditional medium, including mitogen EGF, RSPO, BMP inhibitor Noggin, and Matrigel to generate 3D structures containing all mature cell types [56]. Organoid assay not only proves that Lgr5^+^ cells are indeed ISCs (containing the ability to self-renew and differentiate), but also serves as an important investigation model for regulatory mechanisms of ISCs. More importantly, a variety of organoid models have been developed, which have become important carriers for regenerative medicine and personalized treatment, such as organoid perfusion for refractory ulcerative colitis. After perfusion, wild-type or genetically modified organoids adhere to the injured intestine to reconstruct epithelium. This organoid transplantation therapy provides a potential solution for refractory ulcerative colitis and is in clinical trial stage [57].

## ISCs and colorectal tumorigenesis

CRC is one of the most common tumors in the world. Since its tumorigenic processes correspond to a relatively clear gene mutation feature, CRC has become a classic research model for tumor initiation, and the famous multistep mutation model by Eric R. Fearon and Bert Vogelstein was raised for CRC [58]. It has been demonstrated that intestinal polyps and CRC are significantly similar to normal tissue, all containing typical glandular structures [59]. Moreover, there are heterogeneous cells even in the same tumor, and a small subset of cells express ISC markers and harbor multipotency, which are called CSCs [60–62]. CSCs are pivotal cells in tumor initiation, propagation, drug resistance, and metastasis. The routine strategies to investigate CSCs contain fluorescence-activated cell sorting (FACS) based on surface marker and tumor initiation assay with gradient diluted cells. However, there are some controversies about this assay: (1) Whether a tumor-initiating process of tumor cells in a new environment represents CRC activity *in situ*; (2) The results are largely inconsistent from CSCs with different markers; (3) The cross-talk between tumor cells and immune cells is not considered, as the immunodeficient mice are routinely used for tumor initiation.

### ISCs, CSCs, and tumorigenesis

The investigation of CSCs requires an unperturbed model such as lineage tracing. For an example, “re-tracing” study reveals that Lgr5^+^ adenocarcinoma cells rapidly colonize and differentiate into multi-lineages, which represent self-renewal and differentiation capacities, the two key characteristics of CSCs. Rainbow tracing also validates that Lgr5^+^ tumor cells produce Lgr5^+^ cells and keratin 20 (KRT20)-positive mature cells, and thus have the ability to self-renew and differentiate, conferring Lgr5 as a marker of colorectal CSCs [63]. In recent years, with the advance of technologies such as single-cell sequencing, lineage tracing, and gene knockout, the identification, characteristics, function, and mechanism of CSCs have progressed rapidly, and the CSC model has got accumulated evidences [64, 65]. We believe that the mutation model focuses on molecular events of tumorigenesis, while the CRC model focuses on cellular events. It is necessary to combine these two models together, that is, to investigate the mutation of CSCs, for a better understanding of colorectal tumorigenesis.

**Figure 4.**
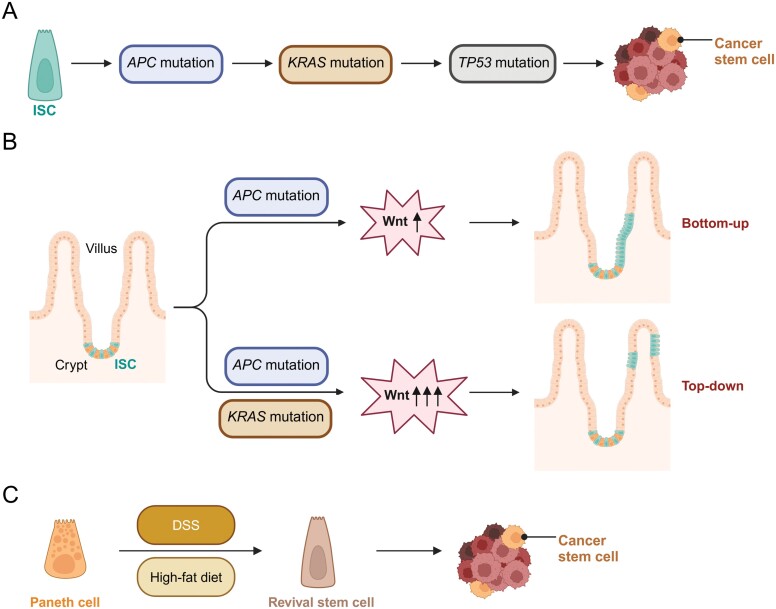
**ISCs, CSCs, and tumorigenesis.**The transformation mechanism from ISCs to CSCs during CRC tumorigenesis, including sequential mutations (A), Lgr5^+^ ISC expansion (B, upper panel), cell-fate reprogramming from Lgr5^−^ non-stem cells (B, lower panel), and Paneth cell dedifferentiation (C). The figures were created with BioRender.com.

Using a lineage tracing model, Nick Barker et al. demonstrate that adenomatous polyposis coli (*APC*) deficiency in Lgr5^+^ stem cells triggers tumorigenesis within 3–5 weeks, whereas *APC* deficiency in villus and transit amplifying (TA) cells rarely induces tumors, probably because of the limited presence period of *APC* deficient cells [66]. It is generally needed for ISCs to mutate several times to transform into CSCs and initiate tumorigenesis, including *APC*, Kirsten rat sarcoma viral oncogene homolog (*KRAS*), tumor protein P53 (*TP53*) mutation, and so on. In this process, the constitutive activation of WNT/β-catenin signaling induced by *APC* mutation is a starting step in colorectal tumorigenesis. *APC* mutation generally leads to the occurrence of adenomatous polyps, and CRCs require additional mutations in other oncogenes or tumor suppressor genes such as *KRAS* and *TP53* (Fig. 4A) [67]. Besides Lgr5, some surface proteins highly expressed on Lgr5^+^ stem cells, such as CD133, have also been identified as markers of colorectal CSCs. CD133^+^ human colon cancer cells, which account for approximately 2.5% of all tumor cells, harbor stronger tumor initiation, self-renewal, and long-term tumorigenic potentials. Accordingly, CD133 is a CSC marker of human colon cancer and mouse small intestinal tumors; however, in mouse colorectal tumors, CD133 is extensively expressed in goblet cells and cannot be used as a CSC marker, indicating the different regional characteristics of the small intestine and large intestine [61, 68].

As mentioned above, accumulating researches validate that colorectal cancer cells originate from ISCs, however, ISCs are apt to differentiate and Lgr5 expression is reduced in tissue damage (like radiation, DSS, and infection) and inflammation (such as IFN-γ, IL-17, and IL-13) state, which are key risk factors for colorectal tumorigenesis. Therefore, several groups have re-evaluated the role of ISCs in inflammation-driven CRC tumorigenesis, including DSS, IBD, or high-fat diet-induced CRC. (1) Inactive mutation of APC or activated mutation of β-catenin induces moderate activation of WNT/β-catenin signaling and expansion of ISCs, but no tumorigenesis. However, a combination of WNT/β-catenin activation and *IkBa*^−/−^ or *Kras* mutation triggers colorectal tumorigenesis efficiently. RelA/p50 and β-catenin/TCF share the same transcriptional activator CBP, thus WNT/β-catenin pathway of *APC*^Mut^;*IkBa*^−/−^ or *APC*^Mut^;*Kras*^Mut^ is hyperactivated, inducing a cell-fate reprogramming from Lgr5^−^ non-stem cells into Lgr5^+^ stem cells, finally driving the occurrence of “top-down” colorectal tumorigenesis (Fig. 4B) [69]. (2) Upon DSS treatment, *APC* knockout in Paneth cells also leads to colorectal tumorigenesis, accompanied by the generation of Paneth cell-derived revival stem cells, a possible mechanism to compensate for reduced Lgr5^+^ ISCs. Moreover, these tumors are consistent with IBD-type CRCs (1%–2%) and even 24.7% human CRCs, which is much higher than IBD-type CRCs. Metabolic inflammation triggered by a high-fat diet causes epigenetic remodeling of Paneth cells to induce dedifferentiation and drive tumorigenesis (Fig. 4C) [70].

These studies altogether reveal that tumor initiation requires mutations in long-lived ISCs, which are not necessarily Lgr5^+^, but can be stem-like cells formed by dedifferentiation of intestinal epithelial cells caused by hyperactivation of WNT/β-catenin, or revival stem cells induced by DSS damage. For simplicity, in the subsequent description of this review, we focus on the transformation of ISCs into CSCs, a classic theory of tumorigenesis currently.

### Cell fate plasticity

**Figure 5.**
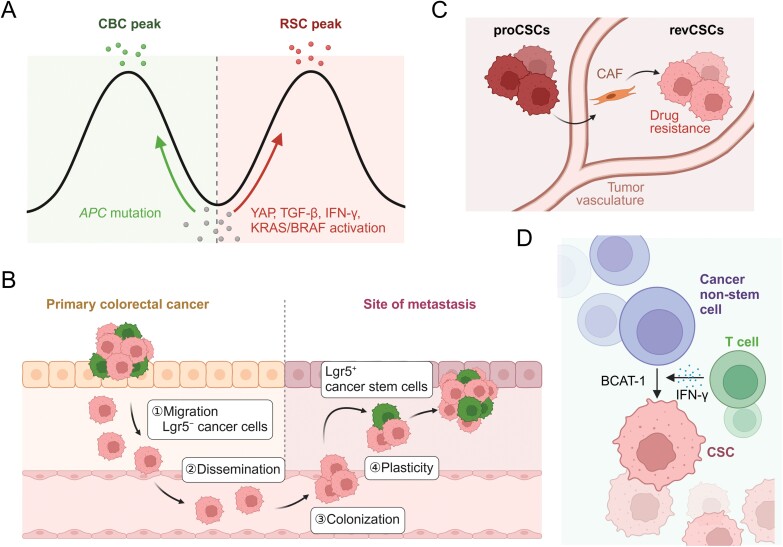
**Cell fate plasticity in multiple CSC states or CSCs–non-CSCs.**Cell fate plasticity in different stem cell status during CRC tumorigenesis (A), Lgr5^−^ non-CSCs and Lgr5^+^ CSCs during CRC metastasis (B), proCSCs to revCSCs during therapy (C), and non-CSCs to CSCs during immunotherapy (D). The figures were created with BioRender.com.

Tumorigenesis process is generally accompanied by the transformation of ISCs into CSCs, during which the state of stem cells changes a lot. Due to the extensive similarities between tumors and embryos, including rapid growth and tissue expansion, vascularization, cell plasticity such as epithelial–mesenchymal transition (EMT), immune tolerance and expression of oncofetal antigens, the fetal-like state of CSCs emerges as one of the most classic characteristics of CRCs [71]. As mentioned above, the original Lgr5^+^ ISCs are lost or differentiated upon DSS, infection, and radiation, and the newly generated ISCs acquire a fate-like state due to activation of IFN-γ and YAP/TAZ. Similarly, CRC tumorigenesis is also characterized by inflammatory response and regeneration, with IFN-γ and YAP/TAZ activation, so it is reasonable to acquire a fetal-like state for ISCs during CRC tumorigenesis. Indeed, Simon Leedham et al. systematically analyze colorectal CSCs with various genotypes and niche, and reveal that there are two types of stem cells in intestinal cancer: Lgr5^+^ stem cells, induced by WNT/β-catenin activation such as *APC* mutation, and Lgr5^−^ regenerative stem cells, which do not exist in the normal intestine but emerges during tumorigenesis. In colorectal tumorigenesis, gene mutations such as *Kras* mutation, V-Raf murine sarcoma viral oncogene homolog B (*Braf*) mutation or transforming growth factor (TGF)-β blockade, and environmental stimulations such as immune factor IFN-γ, stromal TGF-β or matrix Yap signal, induce regenerative stem cells, an alternative stem cell state with fetal-like characteristics. The total stem cell pool is a comprehensive manifestation of two types of ISCs (Lgr5^+^ CBCs and Lgr5^−^ regenerative stem cells). Lgr5^+^ CBCs are enriched in *APC* mutant CRCs, and Lgr5^−^ regenerative stem cells are enriched when YAP, TGF-β, IFN-γ, or MAPK are activated (via Kras or Braf) (Fig. 5A) [72]. In fact, during tumorigenesis, in addition to tumor cells, immune cells such as tumor-associated macrophages and T cells, endothelial cells, tumor-associated fibroblasts and stromal cells all acquire a fetal-like state, forming an “oncofetal ecosystem” [73].

Tumor metastasis is also accompanied by CSC dynamic. Taking advantage of *Vil1*^Cre-ERT2^;*APC*^fl/fl^;*Kras*^LSL-G12D^;*TP53*^KO/KO^;*Rosa26*^Confetti^;*Lgr5*^DTR-eGFP^ mouse model and intravital microscopy imaging technology, Jacco van Rheenen and colleagues demonstrate that CRC metastasis is mainly initiated by Lgr5^−^ cells, and all circulatory tumor cells are Lgr5^−^ cells. With the metastatic locus growing larger, cell-fate conversion occurs from Lgr5^−^ cells into Lgr5^+^ CSCs, which further maintains the hierarchy and long-term growth of metastatic locus via self-renewal and differentiation (Fig. 5B) [74]. Similarly, Lgr5^+^ cell depletion assays also prove the key role of Lgr5^+^ cells in tumor metastasis. In a tumor metastasis model using *Apc*^min/+^;*Kras*^LSL-G12D/+^;*Vil1*^Cre^;*Lgr5*^DTR/eGFP^;*Trp53*^KO^;*Smad4*^KO^ cells, DT treatment depletes Lgr5^+^ cells and largely impairs tumor metastasis [75]. The intestinal regeneration process is accompanied by the release of Hippo’s inhibitory effect on YAP, the inhibition of WNT/β-catenin signaling, and the expression of krüppel-like factor 6 (Klf6), which is collectively termed wound-healing response. Klf6^+^ cells with a wound-healing state inhibit tumor growth and metastasis, and thus, the elimination of YAP/TAZ promotes colorectal tumorigenesis [76]. YAP/TAZ is considered to be carcinogenic in liver cancer. High expression of YAP and TAZ promotes the liver tumorigenesis. *TAZ* knockout alone inhibits liver tumorigenesis, while *YAP* knockout alone has no effect, suggesting the differences between YAP and TAZ at molecular networks as well as the heterogenetic function of YAP/TAZ in different tumors [77]. Mesenchymal-epithelial transition (MET) and high-relapse state are acquired during the colonization of liver metastatic loci from colorectal cancer. Using hepatocyte-specific CRISPRa screening and sLP-mCherry niche labeling system [78], Andreas Moor and colleagues identify that plexin B2 (Plxnb2) expressed by hepatocytes bound to class IV semaphorins (Sema4) on CRC surface, and promotes epithelialization of CRC and liver metastasis [79].

Besides tumorigenesis and metastasis, tumor therapy also induces cell fate dynamics. Almost all clinical drugs, including targeted drugs, chemotherapy drugs, and biological drugs, inevitably lead to tumor resistance, and CSCs play key roles in this process [80]. Researchers have discovered at least two ways for CSCs to resist drugs: (1) A sormant subset of CSCs survives during drug treatment and access into the cell cycle rapidly upon drug withdrawal. Toshiro Sato’s group conducted long-term lineage tracking of individual Lgr5^+^ clones through 4D *in situ* imaging, and divided these clones into dormant state (21%), amplifying state (54%), and regressing state (24%). p27 is highly expressed in dormant CSCs, which resist chemotherapy and mediate tumor recurrence through collagen (COL)17A1 − YAP signaling [81]. (2) Non-CSCs dedifferentiate and regain stemness to fill the lost CSCs, and several works reveal the reprogramming from Lgr5^−^ non-CSCs into Lgr5^+^ CSCs. When Lgr5^+^ CSCs are eliminated with DT treatment, tumor growth is moderately inhibited and recovers rapidly after drug withdrawal [75]. Similarly, the elimination of Lgr5^+^ CSCs with iCaspase 9 and Dimerizer triggers a shrinkage of tumor volume, accompanied by activation of KRT20^+^ differentiated cells and a large number of Lgr5^−^ cells dedifferentiate into colonies. Subsequently, Lgr5^+^ CSCs are obtained again and tumors are expanded. Meanwhile, KRT20 tracing also shows that rare colonies are generated from Lgr5^−^ cells under normal conditions, and a large number of Lgr5^−^ cells dedifferentiate to form colonies upon Lgr5^+^ CSC ablation. Furthermore, Lgr5^−^ colonies are reduced if Lgr5^+^ CSCs are depleted routinely. These observations validate a dynamic reversion of Lgr5^−^ non-CSCs into Lgr5^+^ CSCs and subsequent CRC propagation after Lgr5^+^ CSC depletion [63]. Accordingly, it emerges as a better strategy to target the niche of CSCs than CSCs themselves. Gene fusions of RSPO2 and RSPO3, the ligands of Lgr5, often occur in CRC and are related to hyperactivation of WNT/β-catenin signaling of CSCs [82]. Thus, targeting RSPO3 with antibodies promotes differentiation of CSCs and significantly inhibits the expansion of PTPRK-RSPO3 fused CRC [83].

Tumor therapy not only influences the state of CSCs themselves, as well as their niche. Combined “tumor-fibroblast” organoid co-culture system and 11 therapy strategies, including chemotherapy and signal pathway inhibitors, Christopher J Tape’s group obtained single-cell sequencing data of more than 2500 tumor organoids. Dynamic CSC subsets are observed during tumor therapy, accompanied by vast variations of intracellular signals and post-translational modifications. Specifically, cancer-associated fibroblasts (CAFs) induce CSCs from proliferative stem cells (proCSCs) to revival stem cells (revCSCs) and thus acquire drug-resistant characteristics (Fig. 5C) [84]. Similarly, chemotherapy promotes IL-17A expression in CAFs, further enhancing the stemness of colorectal CSCs [85]. Besides chemotherapy, immunotherapy also leads to variations of CSCs and their niche. One reason for the increased CSC number after immunotherapy is the resistance of CSCs to immunotherapy. Moreover, IFN-γ, a key immune factor of activated T cells in immunotherapy, directly converses non-CSCs into CSCs via a branched-chain amino acid transaminase 1 (BCAT-1)-dependent manner. Therefore, a combination of BCAT-1 inhibitor and immunotherapy efficiently blocks CSC induction by immunotherapy and thus largely improves the effect of immunotherapy (Fig. 5D) [86]. Similarly, another work reveals that type I interferon (IFN-I) produced during tumor immunotherapy drives immune escape and drug resistance. IFN-I induces lysine demethylase 1B (KDM1B) and drives chromatin remodeling, finally triggering the appearance and functional maintenance of CSCs [87]. Of note, the influence of CSCs and their niche are also observed in other tumor types. For example, chemotherapy-induced C5a-GPR77-NFkB activation in breast cancer promotes the emergence of CD10^+^GPR77^+^ fibroblasts, which secrete IL-6 and IL-8 to promote self-renewal of breast CSCs and chemotherapy resistance [88]. The above researches demonstrate that tumor therapy participates deeply in CSCs via disturbances of CSCs themselves and their niches.

### Cell competition

**Figure 6.**
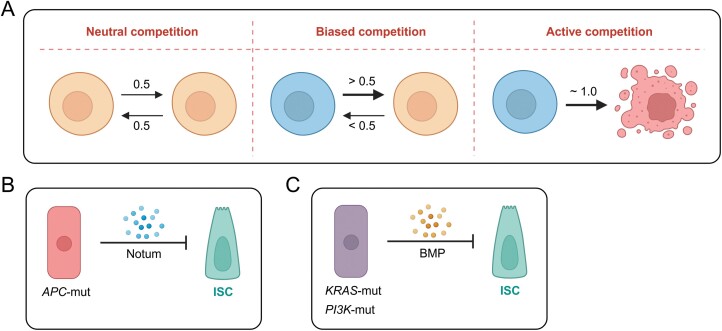
**Cell competition.**Several modes of cellular competition (A), *APC*-mut cells have a competitive advantage (B), and KRAS/PI3K mutations confer a competitive advantage (C). The figures were created with BioRender.com.

Every cell is not alone *in vivo* in multicellular organisms, and cell competition exists all the time, which is an important regulatory factor in the development of multicellular organisms, tissue homeostasis, and tumorigenesis. *APC* mutation is rare in normal intestines, but is common (more than 80%) in CRCs, and thus *APC* mutate cells show remarkable advantages in competition with WT cells. Louis Vermeulen divided cell competition into neutral competition, biased competition, and active competition [89]. Neutral competition is between equipotent stem cells in a limited tissue space, leading to neutral drift in the population. Biased competition is between divergent cells and generally induces an expansion of winner cells and a shrinkage of loser cells. Active competition refers to the winner cells actively disturbing surrounding loser cells, inducing apoptosis, differentiation, phagocytosis, and entosis (Fig. 6A). The differences between winner and loser cells are due to gene mutations, spontaneous disturbances of gene expression, differences in cell adaptation caused by dynamic niche, and positions in the niche. Of note, although winner cells, like *APC*^Mut^ cells, are more likely to obtain competitive advantages, *APC*^WT^ cells sometimes expand.

Two elegant studies show that *APC*^Mut^ cells actively inhibit the expansion of surrounding *APC*^WT^ cells by secreting signals. Using the organoid co-culture model and supernatant transfer model, Louis Vermeulen and colleagues reveal that *APC*^Mut^ cells inhibit the maintenance of surrounding *APC*^WT^ cells and promote their differentiation. *APC*^Mut^ cells secret a variety of WNT/β-catenin inhibitory factors, such as Notum, WNT inhibitory factor 1 (Wif1), and dickkopf WNT signaling pathway inhibitor 2 (DKK2), which induce a differentiation niche and promote the differentiation of surrounding *APC*^WT^ stem cells without affecting *APC*^Mut^ cells themselves. Furthermore, WNT/β-catenin activator LiCl diminishes the competitive advantage of *APC*^Mut^ cells by promoting the overall activation of WNT/β-catenin signaling, and thus significantly reduces intestinal tumorigenesis [90]. Owen J. Sansom and colleagues demonstrate that *APC*^Mut^ cells secrete Notum to inhibit the proliferation of surrounding *APC*^WT^ ISCs and induce *APC*^WT^ differentiation. Notum knockout delays the fixation of *APC*^Mut^ clones and inhibits the occurrence of CRCs. Notum’s inhibitor Notumi also has a similar effect, confirming that *APC*^Mut^ clones gain a competitive advantage by secreting Notum (Fig. 6B) [91]. Similar mechanisms are observed in Kras mutated and phosphoinositide 3-kinase (PI3K) mutated cells. Red2Onco assay, developed to assess the effects of Kras and PI3K activation on cell competition, shows that Kras- or PI3K-activated clones induce a much faster fixation than surrounding WT clones, accompanied by a decrease in stem cell number in surrounding crypts. Mechanistically, *Kras*^Mut^ and *PI3K*^Mut^ clones secrete BMP, which inhibits ISCs directly or indirectly (via remodeling of the stromal cells) (Fig. 6C) [92]. These studies demonstrate that mutant cells can establish a microenvironment that is hostile to normal cells but not hostile to themselves, allowing for their expansion at the expense of normal cells. Of note, these investigations prove that it is a new strategy to diminish the competition advantage of mutant cells by enhancing the function of normal stem cells [93]. At the same time, high expression of Kras, phosphatidylinositol-4,5-Bisphosphate 3-kinase catalytic subunit alpha (PKI3CA) and Rspo3 also promotes the fission of crypt, driving the expansion of mutant cells between different “crypt-villus” units [92, 94, 95].

In addition to gene mutations, heterogeneity in the expression of master genes also induces a competitive imbalance between tumor cells, such as c-Myc expression [96], Yap1 expression [97], and flower (FEW, also known as CACFD1) expression [98]. There is also competition between tumor cells and niche cells for limited space and nutrition, such as the competitive absorption of taurine by SLC6A6^+^ tumor cells and T immune cells [99], the competitive phagocytosis of nutrients and cell debris by Myc^high^ tumor cells and macrophages [100], and the competitive uptake of glutamine by tumor cells and cDC1 through SLC38A2 [101].

### CSC microenvironment

The mutual interaction between tumor cells, including CSCs, and their niche cells plays central roles in tumorigenesis, drug resistance, and metastasis. Among various niche cells, immune cells have emerged as the main component of the success of tumor immunotherapy in recent years. However, CRC has typical immunosuppressive characteristics and resists immunotherapy for most CRC patients [102, 103]. The interaction between CSCs and their niche cells has emerged as a hot topic recently [104].

Tumor cells are regulated by various types of niche cells such as stromal cells and immune cells. Richard Flavell et al. identify a rare type of fibroblasts around crypts via single-cell sequencing. These Prostaglandin-endoperoxide synthase 2 (Ptgs2)-expressing mesenchyme cells are able to process arachidonic acid into PGE2, promote the dephosphorylation, nuclear localization, and transcriptional activity of Yap via PGE2-Ptger4 pathway, and finally drive the expansion of reserve stem cells. Fibroblast-specific *Ptgs2* knockout inhibits the tumorigenesis both in *APC*-deficient and DSS/AOM models, indicating that stromal cells control colorectal tumorigenesis via a paracrine pathway [105]. Accumulating research have shown that various immune checkpoint molecules are target genes of WNT/β-catenin signaling, the most critical pathway in CSCs. Therefore, CSCs constitutively express many immune checkpoint molecules, making them escape immunotherapy easily [106, 107].

Colorectal CSCs also reshape their microenvironment through a variety of mechanisms. Linheng Li’s group reveals the interaction between therapy-resistant CSCs and niche cells using single-cell sequencing. Colorectal CSCs recruit tumor-associated monocytes and macrophages (TAMMs), and TAMMs in turn promote the self-renewal of CSCs through the PGE2–EP4 pathway [108]. Colorectal CSCs also reshape tumor-associated macrophages (TAMs) by exosomal RNA [109]. In order to detect the regulatory effect of niche factors on stem cells in the early stage of colorectal cancer, Ömer H. Yilmaz et al. compare naive AKP (*APC* KO; *Kras*^G12D^; *P53* KO) and *in vivo* AKP tumors, and discover that *in vivo* environment induces AKP cells to a fetal-like state with significantly high expression of Sox17. *Sox17* knockout AKP cells show comparable expansion *in vitro* and in NSG mice but have impaired tumorigenesis in C57BL6 mice compared to AKP cells, accompanied by an increased number of CD8^+^ T cells and activation of IFN-γ, indicating that tumor-intrinsic Sox17 is involved in tumor-immune interactions. Interestingly, Sox17 inhibits the expression of *Lgr5* and interferon-gamma receptor 1 (*IFNGR1*), blocks MHC expression, T cell recruitment and activation via IFNGR1–MHC pathway, and drives cell fate transition of Lgr5^+^ cells to Lgr5^−^ cells for escape from T cell killing [110], which is consistent with previous research that Lgr5^+^ cells are easily killed by T cells [23]. Again, the remodeling of Lgr5^+^ cells into Lgr5^−^ cells induces immune escape and tumorigenesis, echoing cell fate remodeling during tumor metastasis [74].

### CSC macroenvironment

In addition to microenvironment cells, accumulating macroenvironment factors emerge as additional regulatory layers for CSCs and tumorigenesis, including diet, gender, mental stress, dysbiosis, and so on. These newly discovered regulatory networks not only deepen our understanding of CRC tumorigenesis but also provide potential strategies for tumor therapy.

#### Dietary regulation

Food absorption is the entitative function of the intestine, and the regulatory functions of diet and food components on ISCs and CSCs have been widely explored, including high-fat diet [32], calorie restriction [111], high-sugar diet [112] and ketogenic diet [113]. Among them, the molecular mechanisms of a high-fat diet on CRC tumorigenesis are deeply investigated, including: (1) High-fat diet causes metabolic inflammation and the acquisition of a fetal-like state, an important step CRC tumorigenesis [69]. (2) Palmitic acid component in a high-fat diet activates PPAR-γ signaling pathway, making intestinal precursor cells more active to gain tumor-initiating capacity [32]. (3) High-fat diet reduces the diversity of intestinal microorganisms and downregulates of content of Helicobacter. Helicobacter is able to promote the expression of MHC-II through pattern recognition receptor (PRR) and IFNγ signals. Therefore, a high-fat diet induces down-expression of MHC-II in intestinal epithelial cells and ISCs and promotes CRCs through escaping MHC-II-mediated immune surveillance [114]. (4) High-fat diet leads to increased levels of farnesoid × receptor (FXR)-antagonist bile acids, such as T-bMCA and deoxycholic acid, which promote the proliferation of Lgr5^+^ ISCs and CSCs through FXR, and thus FXR agonists can be used to target colorectal CSCs [115].

#### Sexual regulation

 Endogenous gender differences of ISCs are observed in the drosophila model. Females have longer intestines and stronger plasticity of ISCs. Moreover, females harbor more ISCs and stronger proliferation ability after DSS-induced intestinal damage. This sexual difference meets the needs of reproduction for female drosophila but leads to the susceptibility of women to hereditary CRCs [116]. Ronald A. DePinho’s team links genotype and gender differences for the first time. Taking advantage of mouse models and human CRC samples, they demonstrate that only *KRAS* mutant (KRAS*) CRCs exhibit gender differences, while KRAS-WT CRCs do not exhibit gender differences. KRAS–STAT4 pathway targets a Y chromosome gene *KDM5D*, which inhibits the expression of tight junction proteins angiomotin (AMOT) and MHC-I through epigenetic regulation, ultimately driving tumor metastasis and immune escape in a male-specific manner [117]. The critical role of KDM5D in tumor immunity is validated by another research, which reveals that KDM5D and ubiquitously transcribed tetratricopeptide repeat containing, Y-linked (UTY) are responsible for the enhanced immune escape of bladder tumors with LOY (loss of Y chromosome) [118].

#### Microbial regulation

There are various types of microbes in the cavity of intestinal tissue, which emerge as one of the most important environments for ISCs and CSCs. Using ISC-derived organoid formation assay and metabolite screening, Thaddeus Stappenbeck et al. discovered that microbial metabolites generally inhibit the self-renewal and expansion of ISCs, among which butyrate shows the most obvious inhibitory effect. Meanwhile, the intestine has evolved a crypt structure, in which ISCs are distributed at the bottom of the crypt, while absorptive cells at the top absorb and metabolize butyrate efficiently, relieving the inhibition of metabolites on ISCs [119]. However, many specific intestinal microbes promote the stemness of ISCs and CSCs. For example, lactic acid-secreting microbes can act on Gpr81^+^ intestinal stromal cells to promote Wnt3/Wnt2b expression and finally promote the self-renewal of ISCs for intestinal repair after damage [120]. *Faecalibaculum rodentium* promotes ISC proliferation and epithelial turnover via retinoic-acid-eosinophil-IFNγ pathway [121]. Isovaleric acid, a specific metabolite in stools from CRC patients, promotes the expression of tryptophan hydroxylase 2 (*Tph2*) and 5-HT production in intestinal 5-HT^+^ neurons, and 5-HT in turn promotes the self-renewal of ISCs, demonstrating a metabolite–neuron–CSC axis [122]. Recently, an elegant study revealed the role of intestinal bacteria in sexually dimorphic CRC tumorigenesis. *C. maltaromaticum*, significantly depleted in female CRC stools but not males, functions as a tumor suppressor in an estrogen-specific manner. Estrogen drives transcription and expression of *SLC3A2*, the receptor of DD-CPase expressed on *C. maltaromaticum*. Therefore, *C. maltaromaticum* is attached to the gut specifically in females, where they promote vitamin D production by cross-talk with *Faecalibacterium prausnitzii*, and finally VDR signaling is activated for CRC blockade in female [123].

## Summary and prospective

With the development of single-cell sequencing, lineage tracing, and *in vivo* imaging, we have obtained a deeper understanding of ISCs and CSCs, and these efforts have provided potential strategies for clinical therapy. For example, traditional strategy is targeting CSCs themselves, such as cyclooxygenase (COX) inhibitors, which inhibit growth of adenoma cells for CRCs with susceptibility syndrome and sporadic CRCs, but tumor relapse easily after drug withdrwal [124]. Alternatively, it is a potential solution for tumor treatment to target the derailed pathway in dominant mutant stem cells and reduce competitive advantages [91, 93].

However, due to the intertumoral and intratumoral heterogeneity, not all CRCs follow the same hierarchical organization, and accordingly, the proportion, phenotype, activity, potential, and functionality of stem cells in different patients or in different tumor loci of the same patient are inconsistent. The cell composition and distribution characteristics in the CSC niche are also different. Therefore, it is necessary to integrate tumor risk factors such as gene mutations, gene expression perturbations, niche variations, diet, and others in the future, and definitely, uncovering the molecular mechanism of CSCs from a holistic and systematic perspective will provide new strategies to target CRC.
